# Race, Childhood Socioeconomic Status, and Region of Childhood Residence as Intersectional Life-Course Predictors of Cognitive Aging in the United States

**DOI:** 10.1093/geroni/igac020

**Published:** 2022-04-04

**Authors:** Addam Reynolds, Emily A Greenfield, Sara Moorman, Laurent Reyes

**Affiliations:** School of Social Work, Rutgers, the State University of New Jersey, New Brunswick, New Jersey, USA; School of Social Work, Rutgers, the State University of New Jersey, New Brunswick, New Jersey, USA; Department of Sociology, Boston College, Chestnut Hill, Massachusetts, USA; School of Social Welfare, University of California, Berkeley, Berkeley, California, USA

**Keywords:** Brain health, Childhood conditions, Racism

## Abstract

**Background and Objectives:**

Race, childhood socioeconomic status (cSES), and region of childhood residence are each associated with later-life cognition, but no studies have examined how the confluence of these factors influences later-life cognitive performance. Guided by intersectionality theory, we examined individuals’ social positionality across these dimensions as a predictor of cognitive performance in later life among non-Hispanic White (NHW) and non-Hispanic Black (NHB) older adults.

**Research Design and Methods:**

We used data from the 2010–2016 waves of the Health and Retirement Study with participants aged 65 and older in 2010. We employed growth curve modeling to estimate associations among race, cSES, and region of childhood residence, as well as their interactions, and cognitive performance at baseline and over time.

**Results:**

Identifying as NHB, residing in the South, and having lower cSES each were associated with poorer later-life cognition at baseline. Childhood residence in the South was an especially strong risk factor for poorer cognition among NHBs. Among NHWs, higher cSES was associated with better baseline cognitive performance, especially among those from the South. NHBs from the South demonstrated a small advantage of higher cSES, but regardless of cSES, NHBs from the South had lower levels of baseline cognitive scores compared to all other subgroups. We observed steeper declines in cognitive performance over the 6-year study period among participants who lived in the South as children.

**Discussion and Implications:**

Our findings suggest that intersectional social positions across race, cSES, and region of childhood residence primarily influence baseline cognition in later life. Results implicate the importance of attention to multiple life-course social positions in the context of racism within social policies and other initiatives to promote equity in later-life brain health.

**Translational Significance:** Our results underscore the need to tailor life-course brain health initiatives and social policies based on intersectional social positions, which include race, childhood socioeconomic status, and region of childhood residence in the United States. While differences in exposures to risk and protective factors by race/ethnicity and geographic position are widely acknowledged, findings indicate the importance of further orienting to ways in which the effects of risk and protective factors might also differ across intersectional life-course social positions.

Non-Hispanic Black (NHB) older adults are two to three times more likely to experience Alzheimer’s disease and related dementias (ADRD) in comparison to non-Hispanic Whites (NHW; Potter et al., 2009). In the absence of treatment options, the development of prevention strategies to optimize healthy cognitive aging, particularly among those most at risk, is paramount (Smith & Yaffe, 2014). As part of efforts to prevent ADRD, scholars are increasingly interested in identifying early life-course risk and protective factors (Wang et al., 2019). However, life-course scholarship generally has neglected to consider how racism, a fundamental social context that operates independent of socioeconomic status (Phelan & Link, 2015), potentially alters associations between early life-course risk/protective factors and later-life health outcomes (Ferraro et al., 2017), including cognitive performance. This lack of attention might lead to conclusions that identify risk/protective factors as universal across race/ethnicity, while these factors may be moderated by race due to vastly different social contexts stemming from the influence of structural racism.

This study aims to address this gap by focusing on race, childhood socioeconomic status (cSES), and region of childhood residence as early-life antecedents for cognition in later life. We draw on data from the Health and Retirement Study (HRS) to examine how older adults’ intersectional social positions in childhood across these three dimensions of difference are associated with levels of cognition and rates of change over a 6-year period.

## Theoretical Foundation

This study is guided by intersectionality theory. Black feminist and women of color scholars introduced this theory as an analytic framework to explain how multiple categories of social positionality (race, gender, age), as well as systems of oppression (racism, sexism, ageism), interact to (re)produce experiences of discrimination and privilege (Crenshaw, 1989). Region may also be considered as part of intersectional positions, due to differing policy environments throughout the United States that may contribute to systems of oppression. Intersectionality theory rests on the principle that categories of difference are not additive, but simultaneously reinforce each other to produce a unique experience in the context of structural inequality (Bowleg, 2008). Furthermore, some scholars have critiqued an additive approach to the study of historically oppressed groups, given that it assumes that individuals can be ranked by the number of marginalized identities (Bowleg, 2008).

While prior theorizing has discussed cSES and geographic region of residence as potential explanatory factors for racial disparities in cognitive aging (Glymour & Manly, 2008), intersectionality theory motivates our attention to the confluence of these social positions as potentially joint predictors of later-life cognition. Below, we review studies that have addressed cSES and geographic region as singular risk factors for later-life cognition. We also review prior scholarship that considers racial differences in the extent to which cSES and geographic region in childhood influence later-life cognition. We then describe how our study aims to extend this empirical evidence by drawing on intersectionality theory to examine the extent to which the convergence of social positions across race, cSES, and region of childhood is associated with later-life cognition.

## cSES, Race, and Cognitive Aging

cSES is a multidimensional construct reflecting social and economic positions in childhood leading to inequalities due to power differentials across human, social, and financial capital (Vable et al., 2017). Studies consistently have found evidence of the protective effects of cSES for later-life cognition, both in the United States (González et al., 2013; Greenfield & Moorman, 2019; Rogers et al., 2009) and internationally (Zhang et al., 2017). In general, findings have indicated that cSES is associated with baseline cognitive performance, but not change over time (González et al., 2013; Greenfield & Moorman, 2019). Further, these associations are partially mediated by adult SES (González et al., 2013; Greenfield & Moorman, 2019). Evidence also suggests that associations might be specific to cognitive domains (e.g., see Greenfield & Moorman, 2019; Rogers et al., 2009). Scholars have theorized that higher cSES is a protective factor for later-life cognition through multiple life-course pathways, including both sensitive-period effects and social pathways (see Greenfield et al., 2020; Zhang et al., 2020, for discussion).

Largely absent is attention to how race potentially modifies the association between cSES and later-life cognition. Most studies statistically control for race/ethnicity, but do not investigate racial differences as a study aim (e.g., González et al., 2013; Rogers et al., 2009). Other studies only include NHW participants due to sample limitations (Greenfield & Moorman, 2019). One study that has considered racial differences in associations between cSES and later-life cognition found that food deprivation and being thinner were associated with *slower* cognitive decline among Black participants, but did not find any associations among White participants (Barnes et al., 2012). The researchers suggest that these unexpected findings might be attributed to either the benefits of calorie restriction or to selective attrition.

Despite hardly any studies on race as a moderator of associations between cSES and later-life cognition, the theory of Minority Diminished Returns (MDRs) suggests such differences. This theory posits that the protective effect of higher SES is systematically smaller among Hispanic and Black individuals, in comparison to NHWs (Assari, 2018). Scholars have discussed structural barriers to quality education, the labor market, and safe and healthy communities among historically marginalized racial/ethnic groups as mechanisms that might limit the advantages of higher SES to persons of color (Assari, 2018).

Empirical evidence from studies of mental health (Assari, 2014) and mortality (Assari & Lankarani, 2016) supports that the protective effects of higher SES are diminished among individuals from historically marginalized racial/ethnic groups and that this effect is consistent across multiple phases of the life course (Assari, 2020). The literature also supports a transgenerational effect of MDRs on children, with parental education being less influential among Black and Hispanic children in higher SES positions for adolescents’ school performance (Assari, 2018). This body of research supports the importance of examining whether linkages between cSES and later-life cognition differ by race.

## Geographic Position, Race, and Cognitive Aging

Geographic position can refer to specific regions of residence within a larger country, rurality, or another aspect of geographic clustering, such as states with high infant mortality. Geographic position—both current and former—has been associated with cognitive-related outcomes, including risk of dementia (Gilsanz et al., 2019), dementia mortality (Glymour et al., 2011), and stroke (Gilsanz et al., 2017). In particular, Southern residence has been associated with poorer health outcomes across social, physical, and cognitive domains (Glymour & Manly, 2008). However, the definition of what constitutes Southern residence varies greatly by study.

Theoretical accounts on why Southern residence is associated with greater risk of poorer brain health and other outcomes generally have focused on social determinants of health. Such determinants include rurality, socioeconomic disadvantage, poorer nutrition, and less access to technology-based resources (Glymour & Manly, 2008). Glymour and Manly (2008) add to this theorizing by focusing on why Southern residence might be especially detrimental to NHB adults. They posit that “The harm may be disproportionate for [older] blacks … because the functional goal of social organization in the Jim Crow era was to privilege whites at the expense of blacks” (Glymour & Manly, 2008, p. 228). They point to the long-lasting impact of de jure segregation in terms of lower quality and quantity of schooling as contributors specific to lower cognitive performance. They further posit that environmental and institutional harms of being Black in the South can threaten healthy brain aging even among people who migrated away from the South during or after childhood.

There is a growing body of empirical evidence in support of racial differences in childhood geographic position and later-life cognition. Gilsanz and colleagues (2019) found that Black older adults born in states with high infant mortality had a 92% increased risk for dementia, in comparison to White participants born outside this region. In another study, Gilsanz and colleagues (2017) found that older Black adults born in a high-stroke mortality state had the highest risk for dementia, in comparison to non-Black participants born outside of this region. Zhang and colleagues (2016) found that being born in the South was predictive of cognitive impairment at baseline and over time, and that Black older adults were more likely to be born in the South compared to their White counterparts. Esenwa and colleagues (2018) found a dose–response relationship in the association between the density of people who were enslaved in 1860 and stroke mortality in 2011 among Black adults. The authors interpret their findings as indicating that a history of slavery, ongoing social segregation, racial discrimination, and economic inequality are root causes for poor cardiovascular health among Black communities in the Southeastern United States. Taken together, the literature provides preliminary evidence that the association between geographic position and later-life cognition is moderated by race.

## Focus of the Current Study

In this study, we examine how racial identity, cSES, and region of childhood residence potentially jointly influence later-life cognitive performance. The empirical literature reviewed earlier provides a foundation for exploring whether associations between cSES, childhood geographic position, and later-life cognitive functioning differ by race. While scholars have called for increased understanding of the cumulative effect of childhood dis/advantages, including racial differences, no study has examined the confluence of factors, such as cSES and geographical position, within the context of race and later-life cognitive performance (Ferraro et al., 2017). This gap is important to address as most older adults today experienced childhood prior to the passage of the Civil Rights Act of 1964. Accordingly, older NHBs—especially those from the South—experienced Jim Crow era racial segregation during this sensitive period, limiting access to more resourced communities, schools, health care, and other social institutions (Wilkerson, 2011). As the social environment was stratified by race, as well as by SES and geographic position, it follows that risk/protective factors and their associations with later-life cognition might be contingent on these intersectional social positions in nonadditive ways.

Therefore, this study’s research questions (RQs) are as follows: RQ1: Do associations between cSES and later-life cognition differ for NHB adults in contrast to NHW? RQ2: Do associations between residing in the South during childhood and later-life cognition differ for NHB adults in contrast to NHW? RQ3: To what extent do intersectional social positions across cSES, residing in the South during childhood, and race influence later-life cognition?

## Method

### Data

To explore our RQs, we used four waves of data (2010–2016) from the HRS. HRS participants comprise a biennial, longitudinal, nationally representative sample of U.S. adults aged 51 and older. As part of its sampling frame, the HRS includes both primary respondents and their spouses. The study also oversamples Black older adults and those residing in Florida (for detailed information, see Sonnega et al., 2014).

Several selection criteria led to our analytical sample. First, we selected the 2010 wave as our baseline because it was the latest year that the HRS team enrolled new participants, which would maximize the number of NHB participants in our analytical sample. Our first set of selection criteria included having a valid measure of cognition between 2010 and 2016 and being aged 65 or over at baseline. There were originally 10,541 participants that met these criteria; however, after removing those participants without a valid measure of cSES, our analytic sample was reduced to 10,350 participants. Given the nature of our RQs, we removed 1,067 participants who identified as Hispanic or as a race other than White or Black. As SES and geographic region are embedded within national contexts, we further removed from the analytic sample 772 individuals who were not raised or currently residing in the United States. We removed one participant without valid education data. Further, we removed 211 participants (less than 2.5% of the total sample) without valid data on stroke history in 2010. Our final analytical sample included 1,165 NHB and 7,134 NHW participants.

### Measures

#### Dependent variable

The dependent variable was measured using the modified Telephone Interview for Cognitive Status (mTICS), a global measure of cognitive performance (Welsh et al., 1993). The mTICS measure is administered either by telephone or in person, and is scaled from 0 to 35, with higher scores indicating higher cognitive performance. This measure includes subscales of immediate and delayed recall, serial subtraction, object naming, naming the president and vice president, and date identification. Validation of this measure demonstrates an acceptable internal consistency (Cronbach’s alpha: 0.58–0.64; Ofstedal et al., 2005). While a small percentage of participants did not complete this assessment due to refusals, the HRS administrative core has released a multiple imputation strategy across waves that was used in the current study. Consistent with prior literature (Cohen et al., 1999), we transformed raw scores to a percentage of maximum scores to aid in interpretation.

#### Childhood social positions

*Race* is self-reported through a question that asked participants to identify their race. Participants were then asked if they identify as Hispanic or Latino. Using responses to both questions, we created a dichotomous variable to indicate if participants self-identified as either NHW or NHB.

*cSES* was assessed through a validated, multidimensional measure created by Vable and colleagues (2017), encompassing human, social, and financial capital. The human capital subscale is considered an index by combining years of education of both parents. The social capital subscale included items that assessed two factors: maternal investment and family structure. Maternal investment was measured with items that retrospectively asked participants about their mothers’ effort into their upbringing, the amount of time mothers afforded their children, the amount of attention mothers afforded their children, and if mothers taught their children about life. Family structure was assessed by retrospective items that included the number of parental figures, an indicator of whether children lived with their grandparents, and whether children did not live with their mother and/or father. The financial capital subscale included two factors: average financial resources and financial instability. Items included in the average financial resources subscale were self-rated financial status, fathers’ occupation, and an indicator if fathers were unemployed. The financial instability subscale included whether the family received financial help from relatives, whether relocation occurred due to financial constraints, if the family declared bankruptcy, and whether the family lost their business. An index was created by averaging together scores across standardized subscales. Vable and colleagues (2017) used latent variable model estimation to account for missing data and have made the aggregated variable available to researchers on the HRS website. In our analytic sample, scores ranged from −3.57 to 3.06.

*Region of childhood residence* was retrospectively assessed by a single item regarding what state respondents reported living in most of the time during grade school/high school/at the age of 10. The HRS then transformed states of residence into census regions in the publicly available data set. Following the work of Zhang and colleagues (2016), we further collapsed region of schooling into a dichotomous variable indicating if respondents resided in either the South Atlantic (i.e., Delaware, Maryland, the District of Columbia, Virginia, West Virginia, North Carolina, South Carolina, Georgia, Florida), East South Central (i.e., Kentucky, Tennessee, Alabama, Mississippi), or West South Central (i.e., Arkansas, Louisiana, Oklahoma, Texas) Census designations or outside these designations.

#### Covariates

All measures of covariates were based on data collected in 2010. In addition to *age* and *gender,* we created a dichotomous measure of *education* indicating if the respondent had at least 16 years of education, corresponding to a college education. *Current region of residence* was coded in the same way as region of childhood residence (see above). Given potential covariation between stroke history and our focal associations of interest, we also created a dichotomous variable of self-reported stroke history.

### Analytic Strategy

We conducted multivariate analyses using multilevel modeling techniques. We estimated three-level models, wherein observations were nested within participants, who were nested within households. Time was modeled as mean-centered age and mean-centered age-squared. Our analytical approach uses direct maximum likelihood to handle cases with partially missing cognition data (Curran et al., 2010). We first report findings with respect to our RQs for baseline cognitive performance and then for rates of change over time.

Each of the multivariate models included all covariates. The first model examined the main effects of each social position (i.e., race, cSES, region of childhood residence) on baseline cognitive performance and change over time. We then explored our RQs by adding theoretically relevant statistical interaction terms. The final model included a three-way interaction term (cSES × Region of childhood residence × Race), as well as all two-way interactions (cSES × Race, Region of childhood residence × Race, and cSES × Region of childhood residence). To interpret this final model, we estimated marginal linear predictions. We calculated marginal predicted scores on later-life cognition across our four subgroups at 0 ± 1 standard deviation (*SD*) of the cSES variable. Finally, we conducted a series of sensitivity analyses to test the robustness of our findings, attending to our inclusion of covariates and selective attrition.

## Results

Table 1 provides descriptive statistics for all variables across the full analytic sample, as well as four subgroups of participants: NHB from outside the South, NHB from the South, NHW from outside the South, and NHW from the South. These descriptive statistics indicate significant subgroup differences for all study variables. For example, NHB participants from the South had the lowest average cognitive scores at baseline (mean = 49.96; *SD* = 16.65), while NHWs from outside the South had the highest average cognitive scores (mean = 62.99, *SD* = 13.57). NHBs from the South also had the lowest scores on cSES, and NHWs from outside the South had the highest scores. Among the 8,299 participants, 35.26% reported living in the South as children, with NHBs more likely to have resided in the South as children than NHWs.

**Table 1.** Sample Description and Bivariate Test on Key Variables and Covariates

Sample characteristics	Full sample *N* = 8,299	NHB outside South Subsample *n* = 301	NHB from South Subsample *n* = 864	NHW outside South Subsample *n* = 5,072	NHW from South Subsample *n* = 2,062	Subgroup differences^+^
Cognitive performance (2010)^a^	61.01 (14.71)	58.04 (14.10)	49.96 (16.65)	62.99 (13.57)	61.18 (14.55)	η ^2^ = 0.07***
cSES (standardized)	0 (1)	−0.16 (1.00)	−0.53 (0.91)	0.13 (0.97)	−0.07 (1.03)	η ^2^ = 0.04***
Southern childhood residence	35.26%	—	—	—	—	
Black (ref: NHW)	14.04%	—	—	—	—	
Female (ref: male)	58.55%	62.13%	66.09%	56.72%	59.36%	*V* = 0.06***
Age^b^	75.65 (7.16)	74.15 (6.55)	74.65 (6.59)	76.19 (7.33)	75.07 (6.91)	η ^2^ = 0.01***
College (ref: <college education)	21.91%	19.60%	9.49%	24.19%	21.82%	*V* = 0.11***
Current Southern residence	41.56%	16.94%	73.50%	18.45%	88.60%	*V* = 0.64***
Stroke in 2010 (ref: absence of stroke)	11.28%	10.30%	14.35%	10.37%	12.37%	*V* = 0.04**

*Notes*: ANOVA = analysis of variance; cSES = childhood socioeconomic status; NHB = non-Hispanic Black; NHW = non-Hispanic White.

^+^Continuous variables were tested with ANOVA and categorical or binary variables were tested with chi-square.

^a^Percentage of the maximum score.

^b^Age at baseline in 2010.

**p < .01. ***p < .001.

### Multivariate Findings for Baseline Cognitive Performance

Model 1 presents findings from the main effects model. Both NHB participants (*b* = −9.11, *p* ≤ .001) and participants from the South (*b* = −2.84, *p* ≤ .001) had lower average cognitive scores at baseline compared to their counterparts. Moreover, higher levels of cSES were associated with higher baseline cognitive performance (*b* = 1.19; *p* ≤ .001).

To address RQ1, we introduced an interaction term (i.e., Race × cSES), as displayed in Model 2. Consistent with Model 1, higher levels of cSES were associated higher baseline cognitive performance (*b* = 1.21; *p* ≤ .001); however, this association was not moderated by race (*b* = −0.29; n.s.). Similar to Model 1, NHB participants (*b* = −9.32; *p* ≤ .001) and participants from the South as children (*b* = −2.84; *p* ≤ .001) had lower baseline cognitive performance, on average.

To explore RQ2, we introduced the interaction term of Race × Southern childhood residence to Model 1 to produce Model 3. The interaction between Race × Southern childhood residence was statistically significant, indicating that the association between Southern childhood residence and baseline cognitive performance was more negative among NHB participants than among NHW participants (*b* = −0.431; *p* ≤ .001). Consistent with both Models 1 and 2, higher levels of cSES were associated with higher baseline cognitive performance (*b* = 1.18; *p* ≤ .001). Similar to both previous models, NHB participants had lower baseline cognitive performance (*b* = −6.18; *p* ≤ .001).

In Model 4 we included a three-way interaction term, Race × cSES × Southern childhood residence, to address RQ3. This model also included all three two-way interaction terms (Race × Southern childhood residence, Race × cSES, and cSES × Southern childhood residence). Although the three-way interaction term was not statistically significant (*b* = 0.28; n.s.), the model provided consistent evidence of statistically significant two-way interactions. Consistent with Model 3, the interaction between race and Southern childhood residence was associated with lower baseline cognition for NHBs than for NHWs (*b* = −3.95; *p* ≤ .001). In contrast to Model 2, the fully interacted model suggested a statistically significant interaction between race and cSES (*b* = −1.50; *p* ≤ .05). The two-way interaction between cSES and Southern childhood residence also was statistically significant (*b* = 1.75; *p* ≤ .001). Each of the three main effects remained statistically significant and in the anticipated direction as results from Model 1.

**Figure 1.**
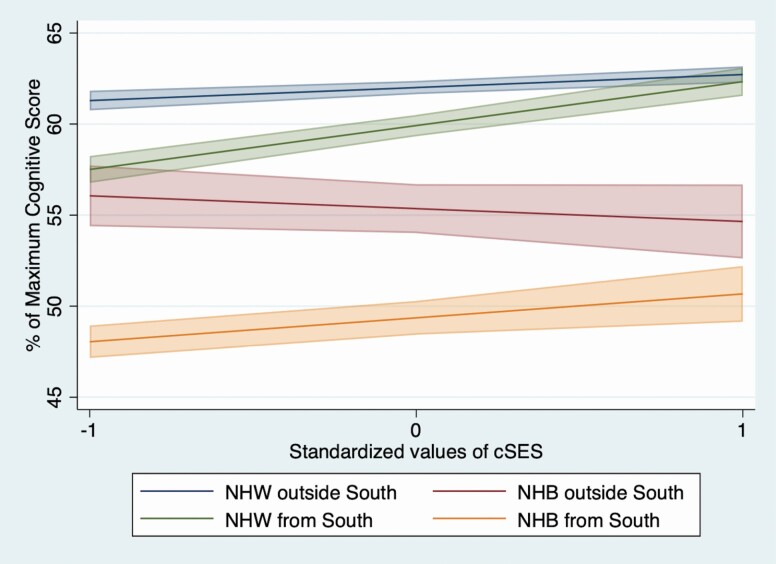
Marginal linear predictions of the interaction between race, cSES, and region of childhood residence. This figure displays the interaction between race, cSES, and region of childhood residence at 0 + 1 standard deviation (*SD*) relative to the sample and total cognition at baseline. *Notes*: cSES = childhood socioeconomic status; NHB = non-Hispanic Black; NHW = non-Hispanic White.

To facilitate interpretation of complex interaction results among the four subgroups, we produced marginal linear predictions based on results from the fully interacted model (Model 4). While typically a decomposition analysis would not be performed in the absence of a significant three-way interaction, the significant results of all of our two-way interactions in Model 4 provided a rationale for performing this analysis. These results suggest differences across the four subgroups requiring further explication, independent of a significant three-way interaction term. Figure 1 demonstrates that cSES buffered against Southern disadvantage among NHW participants, such that at higher cSES scores, NHW participants from the South had similar average scores to NHW from outside the South. In comparison, baseline cognition scores among NHW participants from the South with lower cSES were similar to those of NHB participants from outside the South with lower cSES. However, as cSES scores increase among these subgroups, cSES became less salient for baseline cognition among NHB participants from outside the South. In addition, cSES did not buffer against racial inequities between NHB and NHW participation from outside of the South. Finally, for NHB participants from the South, higher cSES buffered against the Southern disadvantage among NHBs; however, due to the moderating effect of southern childhood residence and race, higher cSES scores among NHB participants from the South did not protect against racialized disadvantage within this regional context.

### Multivariate Findings for Change in Cognition Over Time

Table 2 also presents results with respect to associations across the intersectional social positions and change in cognition over a 6-year period. All four models indicate that being raised in the South is a singular risk factor for steeper declines in cognition over time, with coefficients ranging from −0.11 to −0.15. Only one other statistically significant coefficient emerged across this set of the results. Identifying as Black was associated with greater decline in cognition specifically in Model 2 (*b* = −0.17; *p* ≤ .05). This coefficient was not statistically significant in any other specification of the model. Neither cSES nor race was associated with changes in cognition over time.

**Table 2.** Nested Growth Curve Models Estimating Associations Between Race, cSES, and Region of Childhood Residence With Later-Life Cognitive Performance at Baseline and Over a 6-Year Period

Multilevel growth models	Model 1	Model 2	Model 3	Model 4
Time (age)	−0.68*** (0.04)	−0.68*** (0.04)	−0.67*** (0.04)	−0.68*** (0.04)
Time (age-squared)	−0.01*** (0.00)	−0.01*** (0.00)	−0.01*** (0.00)	−0.01*** (0.00)
Baseline cognition				
Black	−9.11*** (0.40)	−9.34*** (0.44)	−6.18*** (0.70)	−6.76*** (0.72)
cSES (standardized)	1.19*** (0.13)	1.21*** (0.14)	1.18*** (0.13)	0.72*** (0.17)
SCR	−2.84*** (0.36)	−2.84*** (0.36)	−2.21*** (0.38)	−2.17*** (0.38)
Black × cSES		−0.29 (0.39)		−1.50* (0.69)
Black × SCR			−4.31*** (0.84)	−3.95*** (0.90)
cSES × SCR				1.75*** (0.30)
Black × cSES × SCR				0.28 (0.86)
Change in cognition				
Black × Time	−0.11 (0.06)	−0.17* (0.07)	−0.08 (0.10)	−0.14 (0.11)
cSES × Time	0.00 (0.02)	0.02 (0.02)	0.00 (0.02)	0.01 (0.02)
SCR × Time	−0.14** (0.05)	−0.14** (0.05)	−0.15** (0.05)	−0.11* (0.06)
Black × cSES × Time		−0.11 (0.06)		−0.11 (0.10)
Black × SCR × Time			−0.02 (0.13)	−0.05 (0.14)
cSES × SCR × Time				0.07 (0.04)
Black × cSES × SCR × Time				−0.07 (0.12)
Intercept	59.34*** (0.27)	59.36*** (0.27)	59.20*** (0.27)	59.27*** (0.27)

*Notes*: cSES = childhood socioeconomic status; SCR = Southern childhood residence. Standard errors in parentheses. All models control for college education, current Southern residence, and gender.

***p < .001. **p < .01. *p < .05.

### Sensitivity Analysis

In our first set of sensitivity analyses, we evaluated the influence of covariates on results. This analysis is important given that many of the covariates might constitute potential pathways from childhood social positions to later-life cognition. We removed the measure of college education, then stroke, and, finally, current Southern residence. The results of these analyses (available upon request) were the same as those reported earlier in terms of the direction of effects and statistically significant interactions.

In our second set of sensitivity analyses, we introduced additional covariates, such as depression, household income, history of diabetes, smoking history, and a history of high blood pressure. The results were also similar to the results reported earlier.

In our third set of sensitivity analyses, we examined the influence of using the HRS-imputed cognition variable on our results. This sensitivity analysis revealed that approximately 6% of observations were imputed. The results were similar to our main study results, suggesting that the imputed HRS data had minimal influence on our results.

The final sensitivity analysis was conducted to address concerns regarding selective bias for HRS respondents who were at least 65 years old in 2010 and not included in our analytic sample (see *Data* above). There was some evidence of selection bias, with both NHB and NHW participants in the nonanalytic samples having lower average cSES scores in comparison to those in the analytic sample. In addition, the nonanalytic sample included more individuals raised in the South and NHB participants.

To quantify the extent that selective attrition could have biased our results, we performed a simulation analysis. We implemented a multiple imputation strategy using chained equations across 20 data sets using Rubin’s (1987) rules. We imputed the cognitive performance variable across time (i.e., missing at random), stroke history, and education; individuals without any cognition data available were excluded from this simulation. In this analysis, 28% of observations included imputed data and 212 participants were added to our analytic sample. We found similar results across all four models between the imputed and nonimputed data sets. Exceptions included that cSES × Race was associated with steeper declines over time. We further simulated a scenario where the dependent variable was missing not at random by subtracting half an *SD* from the imputed cognition scores as described earlier. This approach was used to provide a worst-case scenario of the influence of selective attrition on study results. Results from Models 1, 2, and 3 were similar using the imputed and nonimputed data sets. In Model 4, the moderating effect of race on the association between Race × Southern childhood residence and baseline cognitive performance was no longer statistically significant. Overall, this not-missing-at-random scenario demonstrates that selection bias might affect some study conclusions.

## Discussion

This study provides evidence that intersectional social positions differentially influence baseline cognitive performance among NHB and NHW older adults. Consistent with findings from prior research (e.g., González et al., 2013; Greenfield & Moorman, 2019), higher cSES was associated with better baseline cognitive performance. However, we found that this association was especially robust among NHWs who resided in the South as children. In fact, higher cSES among NHWs from the South buffered against the Southern disadvantage in baseline cognitive performance, whereby baseline cognitive performance among such individuals at the high end of the cSES scale was statistically similar to those of NHWs from outside the South.

In contrast, among NHBs, higher cSES did not substantially buffer against the cognitive disadvantages of race nor region of childhood residence. Among NHBs from outside the South, the association between cSES and later-life cognitive performance was effectively zero, consistent with the theory of MDR (Assari, 2018). For NHBs from the South, while higher cSES conferred some advantages for later-life cognition, it did not buffer against pronounced disadvantages by racial and geographic position. Our three-way interaction term was not significant, providing evidence that the moderating effect of childhood Southern residence was not further moderated by cSES among NHBs. This lack of significance suggests that being NHB and being raised in the South are driving relative disadvantage independent of cSES. Our results provide mixed evidence of the influence of all three social positions interacting across all subgroups.

We interpret these findings in the context of intersectionality theory, which orients attention to interactions across qualitatively distinct categories of identity and systems of oppression (Crenshaw, 1989). Our methodological approach allowed for the evaluation of multiplicative, rather than additive, categorizations of risk (Holman & Walker, 2021). Our results indicate how additive approaches (i.e., examining each singular risk/protective factor) can lead to an incomplete understanding of the saliency of these factors across populations. For example, if we only considered race, cSES, and region of childhood residence as single risk factors, we would have inappropriately concluded that both cSES and region were equally salient risk factors for NHB and NHW adults. However, our findings with respect to multiple moderating effects underscore the need to conceptualize these early-life social positions as part of matrices of risk and protection.

We found limited evidence for the influence of intersectional positions and rates of change in cognitive performance over time. The only consistent finding was steeper declines in cognition over a 6-year period of later life among participants raised in the South. This association was not moderated by race. These results suggest that Southern social conditions, such as rurality, socioeconomic disadvantage, poorer nutrition, and less access to technology-based resources (Glymour & Manly, 2008), might place individuals at greater risk for cognitive decline in later life. This finding is particularly salient for NHBs from the South, who on average had the lowest baseline cognitive scores across subgroups. Therefore, the ramifications of faster decline may be more immediately noticeable in daily life due to a lower starting position. We conceptualize cognitive decline as different from baseline performance. Decline might indicate increased risk for ADRD, while baseline cognitive performance is likely a stable construct that indicates functional levels across adulthood. However, due to the small size of this effect and the lack of replication of these findings in our sensitivity analysis, we are cautious to overinterpret this result.

While scholars have called for the development of life-course brain health interventions, with targets such as low cSES (Beck et al., 2018), they generally have not oriented to tailoring interventions among those most at risk by intersectional social positions. Our study provides empirical evidence that beyond family-level childhood resources (i.e., cSES), structural barriers that reinforce systems of oppression for NHBs, particularly from the South, are essential to address in the context of cognitive health disparities. Our study demonstrates the importance of dismantling such barriers, such as educational policies, that in multiple ways perpetuate racialized inequalities in cognition, especially among those raised in the South. For example, the racial school achievement gap (Hanushek et al., 2019) is likely an important target for reducing cognitive disparities in later life. Therefore, policies that work towards racial equity in cognitive resources, beginning in childhood, can be considered an important element of healthy brain aging initiatives.

### Limitations

We note several key limitations, especially given our secondary analysis of data from a survey not principally designed as a study of cognitive aging. First, our measure of cognition was limited to a single indicator based on an instrument mainly used as a dementia screener (Welsh et al., 1993). Because more comprehensive assessments of cognition were conducted only with smaller subsets of HRS participants, we were unable to incorporate additional measures of cognitive performance without compromising our analytic sample size, especially among the already limited number of NHB participants raised outside of the South.

Also because of issues of sample size, our study focused exclusively on NHW and NHB adults. It is important for future studies to examine life-course linkages in the context of structural racism among other racial/ethnic groups and other intersectional positions (e.g., transnational migration history). Moreover, as data sets with larger numbers of Black participants become available, especially as they age, future studies can better attend to heterogeneity among this population and within more contemporary cohorts of older adults.

Furthermore, our study drew upon retrospective reports of cSES and residential location, as opposed to administrative or prospective data, raising potential concerns of recall bias. Also, the cSES measure reflected one’s self-report concerning all of childhood, up until the age of 18. It is important for future studies to explore how variations in cSES throughout childhood might affect later-life cognition (e.g., see Last et al., 2018). Moreover, we used a measure of region of childhood residence as a proxy for degree of structural racism due to the relative concentration of anti-Black oppression in the South, especially among older adults. Future studies can include more robust measures that approximate structural racism and geographic differences within U.S. Southern regions, relying on administrative records in geographic contexts (e.g., see Groos et al., 2018). In addition, the measure of region of childhood residence was limited to Census Bureau classifications. Future studies should take a more granular regional approach to facilitate understanding of geographical differences, especially among Southern States. For example, in this paper, while Delaware is technically a South Atlantic state, colloquially and historically it is not considered part of the South (Morris & Monroe, 2009), which could lead to underestimating associations.

Moreover, results from the sensitivity analysis suggested some evidence of the influence of selective attrition. Future studies are needed to replicate these findings, especially using data from studies with more robust longitudinal retention, with a focus on the sustained inclusion of participants from minoritized racial groups.

Finally, it is important for future research to adopt a more explicit strengths-based approach to understanding racial/ethnic disparities in later-life cognition. More holistic depictions of historically marginalized communities and resilience might make more significant contributions to the literature by leveraging the strengths already inherent within these communities (e.g., see Kaup et al., 2015). At the same time, it is important for researchers not to reify that ethnoracial minorities need to do something extra to resist the influence of oppressive forces on later-life cognition. Of further importance is research on cognitive aging that employs methods more explicitly aiming to dismantle racism (for a discussion, see Bowleg, 2021) and its influence on later-life cognition.

## Conclusion

To the best of our knowledge, this study is the first to explore whether intersectional positions across race, cSES, and geographic region of childhood are associated with later-life cognition. We provide evidence that cSES is not as salient a protective factor for baseline cognitive performance among NHB participants compared to NHW participants and that higher cSES is especially protective for the later-life cognitive performance of NHWs from the South. Moreover, the risk of being raised in the South for baseline levels of later-life cognition is especially high for NHB participants, as we found no evidence that higher cSES eliminated the cognitive inequity between NHW and NHB participants in the South. Public health calls from scholars, government agencies, and nonprofit organizations traditionally have focused on individual- or family-level risk factors in adulthood within efforts to promote later-life brain health (e.g., Alzheimer’s Association & CDC, 2018). Notably, the U.S. National Plan to Address Alzheimer’s disease recently added a goal to promote healthy cognitive aging by reducing risk factors for ADRD, including the need to address racial and socioeconomic disparities (Assistant Secretary for Planning and Evaluation, 2021). Our findings emphasize the importance of not only orienting to social determinants of health within brain health prevention and intervention initiatives, but also doing so in ways that consider risk and protective factors across intersectional social positions, including from childhood.
